# Older patients and intensive care: factors that influence patients decisions on life sustaining measures

**DOI:** 10.1186/s12877-026-08315-8

**Published:** 2026-09-22

**Authors:** Adrian Rosada, Drin Ferizaj, Nils Lahmann, Frank Samuel Schaefer, Ursula Müller-Werdan

**Affiliations:** Department of Geriatrics and Medical Gerontology, Charité Universitätsmedizin Berlin, Berlin, Germany

**Keywords:** Life-sustaining measures, Geriatric patients, Attitudes, Ventilation, ICU admission

## Abstract

**Background:**

Physicians treating older patients are frequently unaware of their patients’ personal attitudes towards life-sustaining measures like ventilation, resuscitation, feeding tubes, or ICU admission. In routine clinical practice, there is often little time for reflection prior to deciding in favor or against life sustaining measures in critical medical conditions.

Although decisions for or against these measures should ideally be made jointly by the patient and the treating physician, factors like age, disease burden, social inclusion, and affective state may play an important role in the patient’s motivation.

**Methods:**

Data from 161 inpatients (mean age 82.0 years) on a geriatric ward were collected and analyzed. Patients were interviewed about their attitudes towards life-sustaining measures like ventilation, resuscitation, tube feeding, ICU admission, and dialysis. Four composite indices were formed by combining sign-aligned component variables: medical burden (age and Charlson Comorbidity Index), functional and cognitive impairment (Barthel Index, iADL, and MoCA), affective distress (Geriatric Depression Scale and Hamilton Depression Scale suicidality item), and social support (living situation, having children, and having friends). These indices with other covariates were then analyzed using a Bayesian Rasch item response model with latent regression.

**Results:**

Overall, 14.3% refused all invasive life sustaining measures, whereas 33.8% wished to receive all of the measures listed above. Artificial nutrition was the most refused measure (refused by 53.4%), whereas ICU admission was the most commonly accepted measure (74.4%). Social support had no meaningful association with the decision to accept or refuse life-sustaining measures. Relevant predictors were primarily lower levels of affective distress, and secondarily better functional and cognitive performance and lower medical burden.

**Conclusions:**

Even among older patients, only a minority completely reject life-sustaining measures, even though their life expectancy is often significantly reduced. Patient’s attitude towards life-sustaining measures appears to be related more strongly to affective state, functional performance, and medical burden than living situation or social inclusion. As the patient’s emotional and functional state can certainly be influenced by medical treatment, practitioners should bear this in mind and, where appropriate, reassess their patients’ attitude towards LSM once the aforementioned conditions have improved.

**Clinical trial number:**

Registered with the clinical trial registry Deutsches Register Klinischer Studien.

**Supplementary Information:**

The online version contains supplementary material available at https://doi.org/10.1186/s12877-026-08315-8.

## Introduction

Invasive life-sustaining measures (LSM) such as ventilation, artificial feeding, and dialysis are common in clinical practice. These procedures are used in older patients almost as frequently as in younger patients. However, the probability of survival after treatment decreases significantly with increasing age [1–3]. This illustrates why the term “life-supporting measures” may be more appropriate in the context of older patients than terms such as “life-prolonging measures”. In regard of an aging society and many patients with reduced life expectancy, it is important for physicians to understand patients’ preferences and to treat them according to their wishes.

Previous studies showed that approximately 30% of patients with life-limiting diseases reject interventions such as ventilation or cardiopulmonary resuscitation, yet only a minority had discussed this topic with their treating physician [4, 5]. It has also been shown that when patients discuss these topics with their physician, invasive measures are performed significantly less often, in accordance with the patients’ wishes [6]. Various methods exist to systematically assess treatment limitations in patients who may foreseeably require LSM in the near future [7]. It is currently unclear whether common factors such as age or life-expectancy influence decisions about invasive LSM and whether such factors are broadly applicable. Another study showed that greater frailty and a higher number of comorbidities were associated with a higher likelihood that decisions about limiting LSM were made at all, compared with less frail or less comorbid patients [8].

Patients often present to hospital in critical condition, leaving treating physicians unable to directly elicit their preferences regarding intensive interventions such as ventilation or resuscitation. It has been shown that patients with dementia are more likely to receive LSM than patients without dementia, even when this runs counter to their previously expressed wishes [9].

In these situations, physicians may try to determine the patient’s presumed will by consulting relatives, if available. Such information may be influenced by relatives’ personal attitudes and may diverge from the patient’s wishes [10].

In addition, treating physicians must assess the likelihood that such invasive measures will be beneficial – for example, an older, terminally ill patient eventually may not benefit from ventilation or Intensive Care Unit (ICU) -treatment.

Understanding the conditions that influence these decisions could help clinicians align care with patients’ wishes and tailor treatment accordingly.

Potential determinants of patients’ opinions regarding LSM include age, life expectancy, comorbidities, social inclusion (living with a partner, having children, having friends), affective state, and limitations in functional activities of daily living.

## Methods

Inpatients on a geriatric ward were interviewed by experienced physicians and psychologists about their attitudes towards invasive LSM such as resuscitation, ventilation, artificial feeding, dialysis and ICU treatment. Patients were asked if they have thought about LSM and questions connected with this before, if they like to delegate responsibility for the issue to someone else and if so to whom. This survey was designed to be concise so as not to overwhelm patients. It sought to determine their general attitudes towards certain measures without specifying any time constraints. These LSM did not involve any financial burden for the patients. Before the interview, patients were informed in detail about their medical condition. Patients were required to have sufficient cognitive function to participate in this examination. Therefore, severe cognitively impaired patients were excluded. Additionally, patients were asked about their current living situation, their social inclusion, and their family situation. The compiled questions were incorporated into a questionnaire designed specifically for this study; where possible, established questionnaires or parts thereof were used, supplemented by questions relevant to the specific topic of life-prolonging measures and the emotional context (see Questionnaire in Supplements). Furthermore, a geriatric assessment was conducted to evaluate cognitive function (Montreal Cognitive Assessment (MoCA); range 0 to 30 corresponding to severe cognitive impairment up to normal cognitive findings), affective state (Geriatric Depression Scale (GDS); range 0 to 15; with scores > 5 possibly identify depression; and the Hamilton Depression Scale item on suicidality) [11, 12], functional independence in activities of daily life (ADL) (Barthel Index; 100 indicates complete independence and 0 complete dependence) [13] and in instrumental activities of daily life (iADL) (iADL Index; 8 indicates complete independence and 0 complete dependence) [14]. Patients were classified by reason for hospital admission (falls with or without fractures, infections, or cardiac conditions). For each patient, the Charlson Comorbidity Index was calculated as a surrogate measure of disease burden and estimated life expectancy, with a range from 0 to 37 points and a corresponding estimated 10-year survival of 98% to 0% [15].

### Statistical analysis

We wanted to understand which patient characteristics - such as disease burden, functional decline, depressive mood, or social support - predict the willingness to accept life-sustaining treatments, and to quantify these associations as odds ratios. Because a patient’s answers across the five measures were expected to reflect one shared underlying attitude rather than five independent decisions, we used a joint statistical model that first extracts this common underlying willingness for each patient and then estimates how strongly each characteristic predicts it. Specifically, we used a Bayesian Rasch item response model with latent regression. Each patient is assigned an underlying endorsement propensity, and each treatment has its own threshold reflecting how readily it is generally accepted. The latent propensity was then regressed on four composite indices (medical burden, functional and cognitive impairment, affective distress, and social support) and three additional covariates (gender, self-responsibility in decision-making, desired life expectancy). Each formative index was computed from its component, sign-aligned variables and standardized for comparability. All numeric predictors were scaled using Gelman’s 2-SD scaling to stabilize regularization across predictors measured on different scales. Binary predictors were coded as 0/1 and mean-centered. Models were fit in PyMC 5.27.0 [16] and diagnostic checks were performed with ArviZ [17]. Full details of model specification, prior choices, and posterior predictive checks are provided in Appendix 1. Results are back-transformed and reported as posterior means with 95% highest density intervals (HDIs) on the logit scale. In this context, reported latent-regression coefficients are expressed as odds ratios for a 1 SD increase in each continuous predictor and for a 0-to-1 change in binary predictors. Effects are described as credible when the 95% HDI excludes 0 (equivalently, excludes 1 on the odds-ratio scale). Further supplementary analyses of the primary endpoint under a range of statistical assumptions are provided in Appendix 2.

## Results

### Demographic data

In total, 161 patients were included (mean age 82.0 years; 65.8% female; Table 1).

**Table 1:** Patient characteristics and their functional data

	patients (*n* = 161)
Patient demographics	
Age, years	82.0 ± 6.7
Female sex, n (%)	106 (65.8%)
Charlson Comorbidity Index, mean ± SD	6.2 ± 1.9
Reason for hospitalisation n, (%)	
Fall without/with Fracture/s	58 (36.0%)
Infection (respiratory; urogenital, others)	39 (24.2%)
Cardiac (heart failure, others)	20 (12.4%)
Malignancy	8 (5.0%)
Dehydration	6 (3.7%)
Others (pain, drug intoxication, delirium)	30 (18.6%)
Housing situation n, (%)	
Living alone	101 (63.1%)
Living with partner	47 (29.4%)
Living with children	8 (5.0%)
Living in a nursing home	3 (1.9%)
Children number of, mean ± SD	1.2 ± 1.1
Functional status	
Montreal Cognitive Assessment Score (0 to 30), mean ± SD	25.1 ± 3.2
Activities of Daily Life Score (0 to 100), mean ± SD	39.6 ± 18.6
Instrumental Activities of Daily Life Score (0 to 8), mean ± SD	3.3 ± 1.8
Geriatric Depression Score (0 to 15), mean ± SD	3.8 ± 3.0
Suicidal ideation, Hamilton Rating Scale for Depression (0 to 4; “non-existent” to “all the time”), mean ± SD	0.3 ± 0.6

Data are presented as mean and standard deviation or as number (%), as appropriate

Reasons for hospital admission were diverse. The most common reasons for admission were falls with or without fracture, pulmonary or urinary-tract infections, and congestive heart failure. The mean Charlson Comorbidity Index value was 6.2, corresponding to an estimated 10-year survival of 2% of this cohort resulting from the disease burden. The mean level of functional independence was an ADL-score of 39.6, indicating moderate to severe impairment in activities of daily living (e.g., needing help with mobility, toileting, and eating).

In regard of instrumental activities of daily life, the mean score was 3.3, indicating relevant limitations of the patients (e.g. unable to use any kind of transportation, to do shopping or to run the household). The mean MoCA score was 25.1, indicating generally normal cognitive function and sufficient understanding of the questions. The affective status of the included patients was within the normal range, with a mean GDS score of 3.8 (scores > 5 indicate depressive mood). With regard to suicidal ideation, only a small minority of the examined patients had concrete suicide thoughts (4.5%). In contrast, most patients denied suicidal thoughts, plans, or attempts.

### Social background

Of the participants, 63.1% stated to live alone, 29.4% to live with a partner, 5.0% to live with their children, 1.9% to live in a nursing home. 68.3% reported having children;

76.1% said to have friends with whom they kept in contact.

### Attitude towards invasive life-sustaining measures

Approximately 50% of patients had sufficient cognitive function at the time of the interview to answer questions regarding invasive life-sustaining measures. Patients who did not have sufficient cognitive function were excluded from the interview and final analysis. 35.7% of the patients surveyed stated that they had never thought about LSM, while 64.3% said they had already done so. A majority of 73.1% of patients would prefer to delegate the responsibility for making a decision regarding LSM to someone else. When patients were asked to name someone, who should primarily assume this responsibility, doctors and family members were mentioned with equal frequency.

**Fig. 1:**
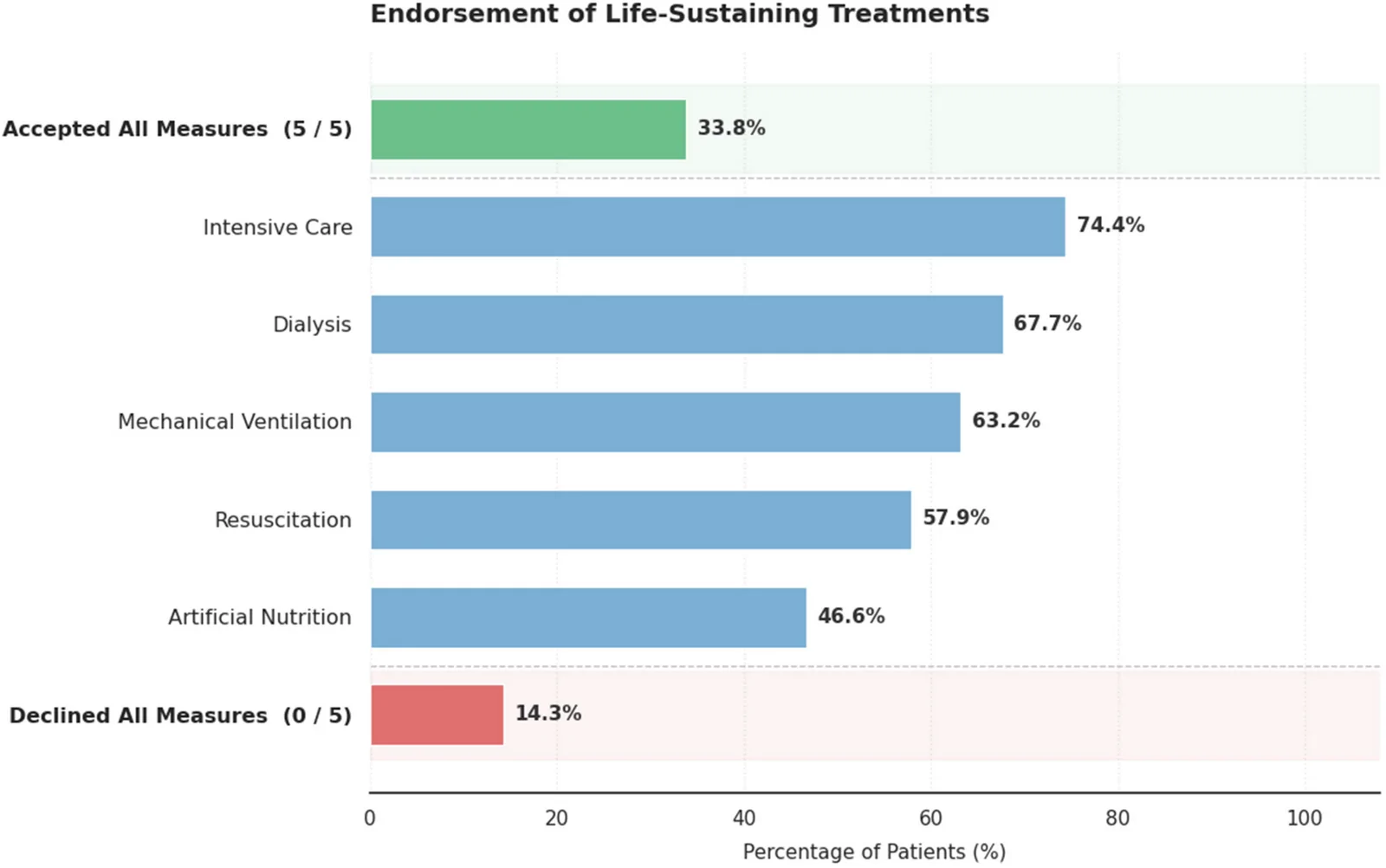
Percentage of patients endorsing specific and general life-sustaining treatments in the complete-case sample

Endorsement rates for all LSM interventions are displayed in Fig. 1. Overall, 14.3% of patients declined all five measures, while 33.8% accepted all five. Across individual items, artificial nutrition was the least accepted (46.6%) and ICU admission the most accepted (74.4%).

### Regression analysis

**Fig. 2:**
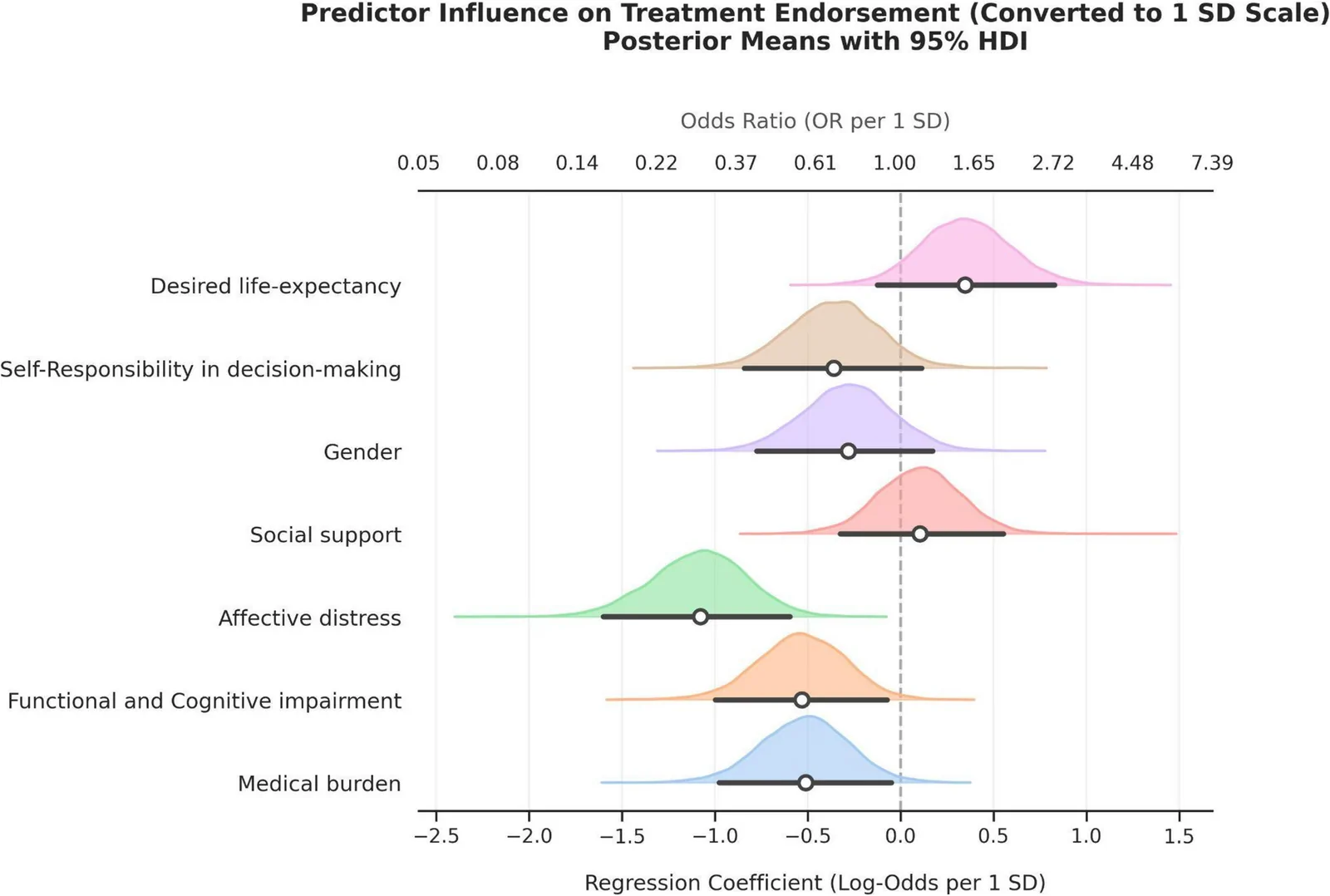
Predictor effects on willingness to accept life-sustaining measures: a Bayesian Rasch latent regression. Note. Posterior distributions of regression coefficients (log-odds scale, bottom axis) and corresponding odds ratios (OR, top axis) for each predictor, scaled to a 1-SD increase in continuous variables and a 0-to-1 change in binary variables. Points denote posterior means; thick bars represent 95% highest density intervals. OR < 1 indicates that higher values of a predictor are associated with lower odds of endorsing life-sustaining measures; OR > 1 indicates higher odds. An effect is considered credible when the 95% HDI excludes OR = 1 (dashed reference line)

The Bayesian Rasch latent regression model (Fig. 2) showed excellent sampling behavior (0 divergences; 0 max-tree-depth hits; BFMI: 0.74 to 0.79; maximum R̂ = 1.001, minimum bulk effective sample size of approximately 4,721; minimum tail effective sample size of approximately 5,935).

A total of 161 patients were included in the primary analysis. Estimated item difficulties reflected clear differences in the general acceptability of the measures. Artificial nutrition had the highest difficulty (*b* = 1.23, 95% HDI [0.77, 1.69]), followed by resuscitation (*b* = 0.34, 95% HDI [− 0.09, 0.79]), ventilation (*b* = − 0.09, 95% HDI [− 0.51, 0.35]), dialysis (*b* = − 0.46, 95% HDI [− 0.90, 0.01]), and ICU admission had the lowest difficulty (*b* = − 1.03, 95% HDI [− 1.51, − 0.57]). Residual heterogeneity in the latent propensity was substantial (SD(*θ)* = 2.56, 95% HDI [2.02, 3.16]), indicating that even after accounting for all predictors, there were large between-patients’ differences in their overall willingness to accept LSM.

Posterior predictive checks indicated that the fitted model reproduced the observed endorsement structure. Posterior predictive checks suggested good model fit, as all observed item endorsement proportions and inter-item correlations fell within their respective 95% posterior predictive intervals, and the posterior predictive mean total score (2.690) closely matched the observed mean (2.693).

In the primary latent regression, higher affective distress (measured with GDS and Hamilton Depression Scale) was associated with meaningfully lower odds of endorsing LSM (OR = 0.334, 95% HDI [0.202, 0.555]), meaning those patients wanted fewer life-sustaining measures. Interpreted on the odds scale, a 1 SD increase in affective distress corresponded to about 67% lower odds of endorsement. Higher medical burden (defined by age & Charlson Comorbidity Index) (OR = 0.597, 95% HDI [0.375, 0.945]) and greater functional/cognitive impairment (OR = 0.580, 95% HDI [0.366, 0.930]) were also credibly associated with lower endorsement propensity, corresponding to ~ 40% and ~ 42% lower odds of endorsement per 1 SD increase, respectively.

Social support showed no credible association with endorsement propensity (OR = 1.110, 95% HDI [0.715, 1.749]).

The remaining covariates showed wide posterior uncertainty, and their effects were not credibly different from zero.

Sensitivity analyses showed that the primary conclusions were robust. Alternative scaling choices and alternative priors, including narrower and wider ridge priors and heavy tailed Student-t priors, yielded highly similar posterior inferences. Furthermore, the full indicator model produced the same pattern in which affective distress indicators, namely suicidality and depressive burden, were the strongest drivers of lower endorsement propensity.

## Discussion

### Principal findings

In this cohort of older, acutely hospitalized patients, preferences regarding invasive LSMs were highly heterogeneous and procedure-specific. A smaller subgroup refused all offered LSMs, while another subgroup endorsed all five. At the same time, endorsements were not uniform across interventions, with ICU treatment being most often accepted and artificial nutrition being least often accepted. Modeling the five binary items jointly supported the clinical intuition that these decisions cluster within persons, but also that patients distinguish meaningfully between specific measures. Against this background of wide baseline heterogeneity, three patient-related domains showed credible associations with a lower overall propensity to endorse LSMs: higher affective distress, higher medical burden, and greater functional or cognitive impairment. By contrast, social support as operationalized here showed no credible association with endorsement propensity, and several additional covariates carried wide uncertainty.

A scenario study found that older adults were less willing to accept LSMs when the scenario involved cognitive (vs. physical) impairment, a worse prognosis, or pain [18]. Our findings extend this pattern to a real inpatient setting, where impairment and prognosis are reflected in current ADL function and comorbidity burden. In our model, both higher medical burden and greater functional/cognitive impairment were linked to a lower overall tendency to endorse LSMs. This fits the idea that many patients weigh LSMs against expected recovery and future independence. At the same time, the large residual heterogeneity shows that health status does not determine preferences on its own. Function and prognosis can help clinicians decide when to start goals-of-care talks and which trade-offs to address, but they should not replace asking patients about their goals.

Prior vignette-based work suggests that end-of-life treatment preferences depend on the scenario and are shaped by psychological factors. It was shown that older adults were more likely to accept treatment in a terminal pain scenario than in a terminal cognitive impairment scenario. Perceived life expectancy was central. People who expected to live longer were less likely to reject treatment, even after accounting for objective health [19]. This links to our results in two ways. First, the large between-person heterogeneity we observed fits with the idea that preferences are not explained by medical burden and impairment alone. Beliefs, values, and expectations likely matter. Second, unlike the vignette study, we found credible associations of higher medical burden and greater functional/cognitive impairment with lower endorsement but no association between preferred life expectancy and endorsement rates. One explanation is the overall context. In the hospital, decisions feel immediate and concrete, so current limitations and prognosis may carry more weight than they do in hypothetical scenarios. This supports the view that preference formation is context-sensitive, and that inpatient data complement community vignette studies.

The strongest association in our data was between higher affective distress and lower LSM endorsement. This finding converges with evidence that depression and suicidal ideation can shape end-of-life attitudes in older medically ill patients. Blank and colleagues, studying hospitalized older adults, found that depression was strongly associated with interest in physician-assisted suicide across scenarios, and that suicidal ideation was a strong predictor of refusals of life-sustaining treatments [20]. At the same time, their work also illustrates an important nuance: depression was only weakly associated with LSM choices in some conditions, and preferences could shift when additional contextual stressors were introduced, such as financial impact [20]. Our study adds to this literature in two ways. First, the association we observe is not limited to a single treatment (such as CPR) but appears as a generalized reduction in endorsement across multiple invasive measures. Second, the size of the association in our data suggests practical urgency: in hospitalized older adults, affective state is a central dimension linked to treatment preferences. Evidence from a dialysis study points in the same direction: in a prospective cohort of 240 hemodialysis patients followed for an average of four years, higher depression symptom levels predicted later withdrawal from dialysis [21].

Depression and suicidal ideation can influence perceived burdensomeness and hopelessness, even when decision-making capacity is intact. A practical clinical response could be to: (1) find out preferences across specific LSM, (2) assess depression and suicidal ideation when feasible, (3) offer symptom-focused support and treatment, and (4) consider revisiting preferences after distress is addressed, particularly when decisions are non-urgent. A similar approach would be appropriate in the case of the functional limitations experienced by those affected, which may still change significantly whilst they are in hospital. Blank et al.’s finding that suicidal ideation predicted LSM refusal underscores why suicidality warrants explicit attention in the clinical workflow [20].

The ordering of interventions in our data argues against compressing goals-of-care documentation into a single yes/no item. It also invites a question that Hui and colleagues addressed: how much of a preference reflects stable values, and how much reflects knowledge, expectations, and framing? In their research, knowledge of life-sustaining procedures was poor, CPR success was often overestimated, and a meaningful subset of participants changed their CPR preference after learning realistic outcomes [22]. These insights can be related to our findings. ICU treatment is a broad category that patients may interpret as a time-limited supportive trial, while artificial nutrition may be understood as prolonged dependency or non-beneficial prolongation. Our findings, together with Hui et al., can support a specific bedside practice: preference elicitation should be paired with brief, standardized, comprehensible descriptions of what each intervention typically entails, including reversibility, burdens, and realistic outcome ranges [22].

In our study, we observed a tendency for female patients to refuse LSM more often which is in line with other studies who also found that women would less likely wish for intensive medical treatment [23–27].

We observed no credible association between our structural social-support index (living situation, children, friends) and endorsement of LSM. However, it should be noted that psychosocial influences include beliefs, expectations, and social roles that go beyond household composition [19]. Our index may not capture relationship quality, perceived burden, or communication patterns. Social context may therefore shape decision processes more than direct yes/no preferences.

Our results underscore the clinical value of documenting a set of treatment-specific preferences rather than relying on a single global decision (e.g., DNR). Prior work shows that preferences differ across interventions and health states, and that functional impairment and depressive symptoms can be associated with a greater tendency to forego treatment in one’s current condition [28]. Together with our findings, this supports structured, intervention-by-intervention conversations (e.g., time-limited ICU trial vs. prolonged ICU dependence; short-term vs. long-term ventilation; dialysis vs. CPR), recognizing that patients apply different acceptability thresholds to each [28]. Finally, the fact that the use of LSM does not depend solely on patients and their attitudes, but also on those who prescribe them, is demonstrated by the following: There are significant regional differences when it comes to the implementation of LSM by physicians: for example, the frequency of withdrawals of LSM is twice as high in Northern Europe as in Southern Europe, which also points to different attitudes and habits on the part of the medical profession [29]. Interestingly dialysis was a measure which a majority (69%) of patients examined in our study wished for, while acute kidney injury with need for dialysis is a very common trigger to consider limiting life-sustaining therapy in older patients for intensive care physicians worldwide [30]. This indicates that there is a significant difference in the perception of LSM between doctors (based on professional knowledge and experience) and patients (based on personal and emotional reasons).

### Limitations

Several limitations have to be considered. Approximately 50 per cent of our screened patients were unable to sufficiently understand the questions regarding LSM due to cognitive impairments and were therefore excluded from the study. This is consistent with the statement mentioned above regarding a significant proportion of critically ill patients who are unable to answer questions on this topic upon admission to hospital and highlights just how useful and helpful a pre-prepared advance care directive is. The assumption is that cognitive impairment is just one factor amongst many that influences attitudes towards LSM, and that the findings from patients without cognitive impairment can, to a certain extent, be extrapolated to the group mentioned above. Our analyses are cross-sectional and cannot establish causal pathways between distress, impairment, prognosis, and preferences [18, 31]. Possible changes during the course of the hospital stay were not recorded. Although Cooper-Kazaz et al. could show in a longitudinal study that attitudes of patient’s relatives towards LSM were almost unchanged after a period of six years, it is not certain whether these results can be transferred to the patients themselves [32]. Ditto et al. could show that older patients presented a “hospitalization dip” directly after hospitalization: 43% of those wishing for LSM before changed their mind to refusal of these measures directly after hospitalization but then changed their mind again towards acceptance of these measures after a period from three months to one year [33]. In contrast, the majority of patients refusing LSM remained firm in their decision at any period (87%). This indicates a certain stability in decisions against these measures, whereas a favorable attitude appears to be rather unstable [33, 34].

Our measures did not directly assess pain, nor did we experimentally manipulate prognostic framing, despite evidence that these dimensions influence preferences [18]. Social support was only captured structurally. In this research, quality of life was not recorded as a specific variable, with the exception of questions relating to depressive symptoms and family circumstances. Finally, stated preferences may not map one-to-one onto documented orders or delivered care during emergencies.

We did not systematically record religiosity, specific socio-cultural or socio-economic factors, so the influence of these factors on attitudes towards LSM could not be investigated. In the context of the existing literature, it can be assumed that religiosity is usually associated with a higher acceptance of these measures [31], whilst a higher level of education tends to be associated with lower acceptance of these measures [35].

These limitations define clear next steps. First, future studies should incorporate explicit measures of pain and standardized prognostic framing to test whether these elements explain additional variance in endorsement propensity [18]. Second, integrating belief measures, especially perceived life expectancy and expected benefit, may clarify how psychosocial mechanisms operate in hospitalized populations [19]. Third, longitudinal designs could test whether within-person changes in distress and function correspond to shifts in treatment preferences, and whether structured information delivery produces more stable, internally consistent preference profiles, echoing Hui et al.’s finding that information can change CPR choices [22].

## Conclusion

In acutely hospitalized very old adults, preferences for invasive LSM vary widely and cannot be reliably inferred from age or social situation alone. Our results align with the established view that impairment and prognosis shape willingness to accept invasive treatment, and they extend it by showing that affective distress is a particularly strong correlate of lower endorsement across multiple LSMs in a real inpatient setting [19, 20]. Clinically, the most practical synthesis is straightforward: discuss multiple interventions rather than a single global decision, provide brief standardized information to reduce expectation-driven distortions, and treat affective distress assessment as part of high-quality, autonomy-respecting goals-of-care practice [21, 22].

## Supplementary Information

Supplementary Material 1.

Supplementary Material 2.

## Data Availability

Due to concerns for participant privacy, data are available only upon request. Please contact Adrian.Rosada@charite.de.

## Abbreviations

ADL: Activities of daily life
BFMI: Bayesian Fraction of Missing Information
CPR: Cardiopulmonary resuscitation
DNR: Do not resuscitate
GDS: Geriatric Depression Scale
HDI: Highest density intervals
iADL: Instrumental activities of daily life
ICU: Intensive care unit
LSM: Life sustaining measures
MOCA: Montreal Cognitive Assessment
OR: Odds ratio
PyMC: Python Monte Carlo
SD: Standard deviation
