# Supplementary material for: Older patients and intensive care: factors that influence patients decisions on life sustaining measures
Source: BMC Geriatr. 2026 Sep 22;26:1211. doi: 10.1186/s12877-026-08315-8 (PMC13615634; doi:10.1186/s12877-026-08315-8)
Supplement: Supplementary file 1 [file 12877_2026_8315_MOESM2_ESM.pdf]

Elderly patients and Intensive Care: Factors that influence Patients decisions on life sustaining measures - “LEBENSMUT”

Questionnaire about Patients wishes for life sustaining measures in context of depression, life weariness and social situation

Version 1.3

12 December 2024

Charité Universitätsmedizin Berlin, Department of Geriatrics and Medical Gerontology

This questionnaire examines attitudes towards potential medical interventions, the current living situation and the mental well-being of geriatric patients. It takes approximately 15 minutes to complete. The questionnaire must be administered by an experienced and trained interviewer, and all questions should be answered as fully as possible.

Date of examination **Initials of the investigator**

Part 1-Patients personal data

Name:

1. **(Main) reason for admission to hospital**

   infection
   fall without fracture
   fall with fracture
   dehydration
   malignant disease
   heart disease
   others

2. **Do you have children of your own?**

   Yes / No

   a. **How many?**

   b. **Do these children live nearby?**

   Yes / No

3. **What is your current living situation? Are you currently**

   living alone,
   with a partner,
   with children,
   in a care home,
   in some other arrangement?

4. **Do you have active friendships?**

Part 2-Wishes of Patients in regard of medical treatment[1]

1. **Please imagine that you could no longer eat on your own. If there were no other option, would you want to be fed artificially (e.g. via a nasogastric tube or parenteral nutrition)? (What do you understand by this?)**

   Yes / No

2. **If your heart were to stop beating, would you want to be resuscitated (by means of chest compressions, artificial respiration and, if necessary, a defibrillator)?**

   Yes / No

3. **If you were no longer able to breathe on your own, would you want to be put on a ventilator (with an oxygen mask or a tube (in the airways))?**

   Yes / No

4. **If your kidneys stopped working, would you want to undergo renal replacement therapy (dialysis)?**

   Yes / No

5. **If you were so ill that you needed to be transferred to an intensive care unit, would you agree to that?**

   Yes / No

6. **Have you ever given any thought to questions like these?**

   Yes / No

7. **Would you prefer to take responsibility for such matters yourself (or leave it to a doctor, family members or others)?**

   Doctor **family member** **other:**

Part 3- depressive mood, suicide ideation and weariness of life

1. **Geriatric depression scale (GDS-15) completed with the patient?[2]**

   Yes / No

2. Suicide ideation?[3]

0 |__| Absent.
1 |__| Feels life is not worth living.
2 |__| Wishes he/she were dead or any thoughts of possible death to self.
3 |__| Ideas or gestures of suicide.
4 |__| Attempts at suicide

3. Wish to live/ wish to die?[4]

a. My will to live is:
non-existent to particularly strong

1 2 3 4 5 6 7

b. My desire to die is:
non-existent to particularly strong

1 2 3 4 5 6 7

Part 4-general questions

1. If you could choose, how old would you like to live to be?

a. Why this age?

2. What important lessons have you learnt in your life?

3. Is there anything in your life that you regret?

Yes / No

a. If so, what?

References

1. **Items adapted from the Therapy Limitation Documentation Form-**
   Neitzke, G., Böll, B., Burchardi, H. et al. Dokumentation der Therapiebegrenzung. Med Klin Intensivmed Notfmed 112, 527–530 (2017). https://doi.org/10.1007/s00063-017-0321-x
2. **Yesavage JA, Brink TL, Rose TL, Lum O, Huang V, Adey M, et al. Development and validation of a geriatric depression screening scale: a preliminary report. J Psychiatr Res. 1982;17(1):37–49.**
3. **Item adapted from Hamilton Depression Rating Scale (HAM-D)-**
   Hamilton M. Rating depressive patients. J Clin Psychiatry. 1980;41(12 Pt 2):21–4.
4. **Items adapted from Suicide Status Form II (SSF-II)_G -**
   Conrad, A. K., Jacoby, A. M., Jobes, D. A., Lineberry, T. W., Shea, C. E., Arnold Ewing, T. D., & Kung, S. (2009). A psychometric investigation of the Suicide Status Form II with a psychiatric inpatient sample. Suicide and Life-Threatening Behavior, 39(3), 307–320
