# Supplementary material for: Older patients and intensive care: factors that influence patients decisions on life sustaining measures
Source: BMC Geriatr. 2026 Sep 22;26:1211. doi: 10.1186/s12877-026-08315-8 (PMC13615634; doi:10.1186/s12877-026-08315-8)
Supplement: Supplementary file 2 [file 12877_2026_8315_MOESM1_ESM.docx]

**Appendices**

**Appendix 1: Detailed Specifications of the Bayesian Item Response Models**

**A1.1 Predictor Construction for the Primary Model**

We modelled five binary life-sustaining measure (LSM) items jointly using fully probabilistic Bayesian models fit in PyMC 5.27.0 with NUTS (No-U-Turn Sampler / Hamiltonian Monte Carlo). All posterior inference was summarized with ArviZ 0.22.0. In plain terms, the model first estimates each patient’s overall tendency to say yes to invasive, LSM treatment, and then aims to find out which patient characteristics shift that tendency up or down. The following five binary outcome variables (0 = decline; 1 = accepts) were selected:

```python

OUTCOME_ITEMS = [

"artificial_nutrition", # least accepted overall

"resuscitation",

"ventilation",

"dialysis",

"icu_treatment", # most accepted overall

]

```

Some clinical variables were assembled into composite indices before entering the models. Each index is formed by (a) sign-aligning its component variables so that higher values uniformly mean more of the construct, (b) averaging the aligned components, and (c) re-scaling the result to a mean of 0 and a standard deviation of approximately 0.5 (Gelman's 2-SD transformation). Reported odds ratios for continuous predictors are back-transformed to represent a +1 SD increase in the original variable: OR = exp(β/2). Standalone continuous predictors receive the same 2-SD scaling. Additionally, binary predictors (sex, self-responsibility in decision-making) are coded 0/1 and mean-centred. The desired life-expectancy variable (reported in years) is log(1+x)-transformed before scaling to reduce potential right skew. The primary predictor set uses four indices plus three standalone terms:

Table A1.1 Indices Composition

| **Domain** | **Indicators and sign** |
| --- | --- |
| Medical burden | Charlson Comorbidity Index (+), Age (+) |
| Functional/cognitive impairment | MoCA(−), Barthel ADL (−), Instrumental ADLs (−) |
| Affective distress | GDS (+), Hamilton suicidality item (+) |
| Social support | Social embeddedness (+), number of children (+), children nearby (+), friendships (+) |

```python

\# ── Predictor configuration ─

\# Sign convention:

\#	+1 means higher raw value → higher index score

\#	-1 means higher raw value → lower index score (reversed)

PREDICTOR_CONFIG = {

\# INDICES (components averaged then rescaled)

"medical_burden": {

"Charlson_Comorbidity_Index": +1,

"age": +1,

},

"functional_cognitive_impairment": {

"Barthel_ADL": -1, # low score = more impaired

"iADL": -1, # low score = more impaired

"cognitive_screening_score": -1, # low score = more impaired

},

"affective_distress": {

"GDS_depression": +1,

"Hamilton_suicidality": +1,

},

"social_support": {

"social_integration": +1,

"number_of_children": +1,

"children_nearby": +1,

"friendships": +1,

},

\# STANDALONE (entered to the model individually)

"sex": "binary”, # mean-centred

"self_responsibility": "binary”, # mean-centred

"desired_life_expectancy": “continuous”, # log1p transformed and 2-SD scaled

}

```

Additionally, several model and index configurations (e.g., three domains from collapsing medical burden and functional impairment or social variables entered individually) were used as sensitivity analyses and are reported in Appendix 2.

**A1.2 Primary Model: Bayesian one-parameter Rasch latent regression**

This is a one-parameter logistic (Rasch) model in which each person's overall willingness to endorse LSMs is captured by a latent trait θ, and each of the binary outcome items has its own difficulty b. The key idea is that the five item responses are not treated as independent endpoints: they are connected through the shared latent trait. Therefore, a patient who accepts ICU admission is more likely to also accept resuscitation. To test these assumptions, we first examined whether the five binary LSM items were consistent with a single underlying latent dimension. An exploratory factor analysis based on tetrachoric correlations and maximum likelihood extraction supported unidimensionality. The item set showed good factorability, with KMO = 0.833 and a highly significant Bartlett’s test of sphericity, χ²(10) = 201.13, p < .001. All five items loaded positively on the same factor, with loadings ranging from 0.601 to 0.745 (ICU admission = 0.745, resuscitation = 0.728, artificial nutrition = 0.694, mechanical ventilation = 0.680, dialysis = 0.601). The first eigenvalue clearly dominated the remaining eigenvalues (2.904, 0.665, 0.555, 0.474, 0.402), and the one-factor solution explained 47.8% of the total variance. Pairwise inter-item correlations were uniformly positive, ranging from 0.39 to 0.58.

The latent trait for each person is regressed on the predictor indices:

*θ_p_* = *β₀* + *X_p_* · *β* + *σ_θ_* · *z_p_	z_p_* ~ Normal(0, 1)

*y_pi_* ~ Bernoulli( logistic(*θ_p_* − *b_i_*) )

Here, *θ_p_* is the latent willingness of person *p* to accept LSMs. *β₀* is the intercept, *X_p_* is the vector of predictor values for person *p*, *β* is the vector of regression coefficients, *σ_θ_* is the residual standard deviation of the latent trait, *z_p_* ~ Normal(0, 1) is the person-specific residual term, *b_i_* is the difficulty of item *i*, and *y_pi_* is the observed response of person *p* to item *i*.

Item difficulties are sum-to-zero constrained so that β₀ is interpretable as the population average log-odds of endorsement. Reported regression coefficients (β) are on the logit scale. They are converted to odds ratios for reporting.

**A1.3 Priors and Sampling Settings**

A prior is the probability distribution assigned to a parameter before the current data are taken into account. In practice, priors in this setting function as regularization: they stabilize estimation and make implausibly large effects less likely unless the data strongly support them. All priors are weakly informative and regularising. The key choice for regression coefficients is a hierarchical ridge prior: each β_k is drawn from Normal(0, σ_β), where σ_β itself has a HalfNormal(0.5) prior. Because predictors are 2-SD scaled (SD ≈ 0.5 after scaling), the prior effectively places most mass on odds ratios below ~exp(1) ≈ 2.7 per predictor unit while remaining adaptive. For the case that the data supports a larger effect, σ_β grows to accommodate it. For item difficulties, a Normal(0, 1.5) prior was used on the log-odds scale. This places most prior mass on probabilities between roughly .05 and .95 while allowing the data to speak if a strong signal were to occur.

´´´python

\# ── Prior configuration ───

DEFAULT_PRIORS = {

\# Item difficulties on a logit scale

"b_sigma": 1.5, # Normal(0, 1.5) on each item difficulty

\# Regression intercept (population mean log-odds of endorsement)

"beta0_sigma": 1.5, # Normal(0, 1.5)

\# Regression coefficients with an hierarchical ridge

"beta_k": "Normal(0, sigma_beta)",

"sigma_beta": "HalfNormal(0.5)", # ridge scale; data-adaptive shrinkage

\# Person-level heterogeneity (residual SD of theta after regression)

"sigma_theta_scale": 1.0, # HalfNormal(1.0)

}

\# ── Sampling configuration ──

SAMPLER = {

"draws": 2500,

"tune": 2500, # warm-up steps (discarded)

"chains": 4,

"cores": 4,

"target_accept": 0.95, # NUTS dual-averaging target

"max_treedepth": 15,

"init": "jitter+adapt_diag",

"random_seed": 42,

}

´´´

**A1.4 Model Checking**

Convergence was assessed via R-hat (all values < 1.01) and effective sample size (bulk and tail ESS > 4,000 for all scalar parameters in the primary model). No divergent transitions and no max-tree-depth hits were observed across any run. Bayesian Fraction of Missing Information (BFMI) was 0.70–0.79 across chains.

Posterior predictive checks were used to evaluate fit. We verified that:

- Per-item endorsement proportions predicted by the model covered the observed proportions within their 95% posterior predictive intervals.
- The predicted distribution of total LSM scores (0–5) matched the observed distribution, including the mean (predicted: 2.690; observed: 2.693).
- Pairwise inter-item correlations fell within their 95% posterior predictive intervals.

Model comparison used PSIS-LOO (Pareto-smoothed importance sampling leave-one-out cross-validation) at the response level. Pareto-k diagnostics indicated that a small number of observations had moderate leverage (k > 0.5: ~30 items in the GLMM; mostly driven by the clustered within-person structure), with no observations exceeding k > 1.0.

**Appendix 2: Sensitivity and Robustness Analyses**

A2.1 Overview and Rationale

The main manuscript reports a Bayesian Rasch latent-regression model as the primary analysis. This appendix aims to address a narrower question: do the conclusions depend on specific analytic choices, or do they persist across other defensible modelling specifications? Therefore, we followed the logic of multiverse analysis where we varied potential and reasonable analytical decisions to examine the robustness of the primary findings (doi: 10.1177/1745691616658637). In particular, we varied three central aspects of the primary analysis:

1. Prior and scaling assumptions around the primary Bayesian Rasch model,
2. Predictor representation (how the underlying variables were grouped into domains), and
3. Model family / estimand (subject-specific latent models vs. marginal population-averaged models; Bayesian vs. frequentist estimation).

Across these different branches, the same pattern remained visible: greater affective distress was the most consistent predictor of lower willingness to accept life-sustaining measures (LSM). When overall vulnerability was split into subdomains, medical burden and functional/cognitive impairment both showed independent negative associations. Additionally, social support did not show a relevant association with the willingness to endorse LSM.

A2.2 Shared analysis conventions

All robustness analyses used the same basic conventions as the primary model in Appendix 1. In particular:

- The same five binary LSM items were analyzed jointly: artificial nutrition, resuscitation, ventilation, dialysis, and ICU admission.
- The same sign conventions were retained throughout, so that higher values always indicated more of the named construct.
- Covariate missingness was low (approximately 1% to 2%) and was handled using Bayesian imputation in PyMC.
- Unless otherwise stated, Bayesian models followed the same software and sampling framework as Appendix 1 (PyMC 5.27.0, ArviZ 0.22.0, No U-Turn Sampler).
- In the Bayesian models, continuous predictors used Gelman’s 2-SD scaling. However, reported ORs for continuous predictors were back-transformed to represent a +1 SD increase in the original variable (OR=exp(β/2)). Binary predictors are reported as the 0-to-1 OR (OR=exp(β)).
- In the frequentist replications, corresponding predictors were z-standardized, so ORs for continuous predictors are directly interpretable as per +1 SD.

A2.3 Sensitivity to prior and scaling choices around the primary Bayesian model

Bayesian inference can, in principle, be influenced by the choice of prior and by the scale on which predictors are entered. We therefore first varied these assumptions for our primary 4-domain Rasch latent-regression model to test whether the main conclusions were robust to different regularization choices.

*Table A2.1*
*Prior and scaling sensitivity analyses around the primary Bayesian Rasch model*

| **Branch** | **Change relative to the primary model** | **Rationale** | **Substantive** “perfect solution”. We therefore tested whether the conclusions depended on particular **conclusion** |
| --- | --- | --- | --- |
| Alternative scaling  with hierarchical ridge: σ_β ~ HalfNormal(0.5) and β_k ~ Normal(0, σ_β) | Replaced 2-SD scaling with conventional z-scoring and prior widths adjusted accordingly | To check whether the Gelman 2-SD scaling convention itself drives the results | Primary conclusions were replicated |
| Narrower ridge prior with σ_β ~ HalfNormal(0.3) and β_k ~ Normal(0, σ_β) | Stronger shrinkage on regression coefficients | To check whether the core effects survive more aggressive regularization | Primary conclusions were replicated |
| Wider ridge prior  with σ_β ~ HalfNormal(1.0) and β_k ~ N(0, σ_β) | Weaker shrinkage on regression coefficients | To check whether the observed effects are artifacts of over-shrinkage | Primary conclusions were replicated |
| Student-*t* priors with σ_β ~ HalfNormal(0.5), β_k ~ Student-t(ν = 4, 0, σ_β) | Replaced Gaussian priors with heavier-tailed priors | Allows occasional larger effects if strongly supported by the data; checks robustness to outlying coefficients | Primary conclusions were replicated |
| Regularized Horseshoe  with 3 expected non-zero effects and τ ~ HalfNormal(0.022), λ_k ~ HalfCauchy(1), z_k ~ Normal(0, 1), and c^2 ~ InverseGamma(2, 2), with β_k defined through the regularized horseshoe construction | Replaced the ridge prior with a regularized horseshoe prior on the regression coefficients | To test whether the main findings persist under a flexible prior that combines global shrinkage of coefficients overall with local flexibility that can allow a small number of effects when the data shows strong effects | Primary conclusions were replicated. |

*Note*. β_k denotes the regression coefficient for predictor k. σ_β is the global scale parameter governing the overall amount of shrinkage in the ridge prior. In the Student-t priors, ν is the degrees-of-freedom parameter, with smaller values indicating heavier tails and larger ν indicating a prior closer to normal. In the regularized horseshoe specification, τ is the global shrinkage parameter, λ_k is the local shrinkage parameter for coefficient k, z_k is an auxiliary standard normal parameter, and c^2 defines the slab component that prevents unrealistically large coefficients.

Across all branches, the ordering and direction of the main effects did not change: affective distress remained the strongest negative predictor; in the split-frailty specification, medical burden and functional/cognitive impairment remained negative; and social support remained uncertain.

We also explored more flexible 2PL item-response variants, which relax the equal-discrimination assumption of the Rasch model. In the present sample, however, these models did not achieve stable convergence and were therefore not interpreted further.

A2.4 Examining different indices and predictor sets

The next step was to vary how the predictors were represented. The formative grouping of variables into indices is often reasonable and practical, but there is no such thing as a domain definitions.

*Table A2.2*
*Predictor-set variations used in the robustness analyses*

| **Specification** | **Predictor representation** | **Substantive question** |
| --- | --- | --- |
| 3-domain model | Domains: Broad frailty/vulnerability (consisting of medical burden + functional/cognitive impairment) + affective distress + social support  Covariates: gender + self-responsibility + desired life expectancy | Is one broad vulnerability construct sufficient? |
| 4-domain model (primary model) | Domains: Medical burden + functional/cognitive impairment + affective distress + social support Covariates: gender + self-responsibility + desired life expectancy | Does splitting frailty into clinically distinct subdomains add information? |
| Social-as-single-indicators model | Domains: Broad frailty/vulnerability + affective distress  Covariates: individual social variables + gender + self-responsibility + desired life expectancy | Does a social composite hide opposing component effects? |
| Full indicator decomposition | All original predictors entered separately | Do the domain scores create or conceal any signal? |

The 4-domain model was retained as the primary specification in the manuscript because it offers the clearest clinically interpretable decomposition of vulnerability while preserving the same substantive conclusions seen in the simpler and more disaggregated variants.

A2.5 Bayesian subject-specific models: Rasch latent regression across predictor sets

The central robustness analysis kept the subject-specific Rasch latent-regression framework and varied only the predictor representation. Thus, these models ask: how do patient characteristics shift a person’s underlying willingness to endorse invasive LSM treatment, after accounting for stable between-person heterogeneity and item difficulty?

*Table A2.3*
*Bayesian Rasch latent-regression results across predictor specifications*

| **Specification** | **Predictor** | **OR** | **95% HDI** |
| --- | --- | --- | --- |
| **3-domain model** | Frailty/vulnerability | 0.45 | [0.28, 0.74] |
|  | Affective distress | 0.33 | [0.20, 0.53] |
|  | Social support | 1.11 | [0.72, 1.72] |
|  | Gender | 0.55 | [0.20, 1.37] |
|  | Self-responsibility | 0.48 | [0.18, 1.32] |
|  | Desired life expectancy | 1.45 | [0.90, 2.31] |
| **4-domain model (primary)** | Medical burden | 0.60 | [0.37, 0.95] |
|  | Functional/cognitive impairment | 0.58 | [0.37, 0.93] |
|  | Affective distress | 0.33 | [0.20, 0.56] |
|  | Social support | 1.11 | [0.71, 1.75] |
|  | Gender | 0.56 | [0.22, 1.50] |
|  | Self-responsibility | 0.48 | [0.17, 1.24] |
|  | Desired life expectancy | 1.43 | [0.89, 2.30] |
| **Social-as-single-**  **indicators model** | Frailty/vulnerability | 0.46 | [0.29, 0.76] |
|  | Affective distress | 0.33 | [0.19, 0.54] |
|  | Gender | 0.55 | [0.22, 1.40] |
|  | Self-responsibility | 0.50 | [0.19, 1.30] |
|  | Desired life expectancy | 1.43 | [0.90, 2.25] |
|  | Social embeddedness | 1.30 | [0.81, 1.97] |
|  | Number of children | 1.18 | [0.75, 1.89] |
|  | Children living nearby | 0.86 | [0.55, 1.36] |
|  | Friendships | 0.94 | [0.60, 1.48] |

**Note.** Continuous predictors in the Bayesian models were entered on the 2-SD scale; ORs in this table have been back-transformed to represent a +1 SD increase in the original predictor: OR = exp(β/2). Binary predictors are reported as the 0-to-1 OR: OR = exp(β). Intervals are 95% highest density intervals (HDIs).

In summary, the sensitivity analyses replicated the primary findings. In this context, affective distress was the most stable and strongest negative predictor in every specification. This result was not contingent on whether vulnerability was represented broadly or split into medical and functional/cognitive indices. Furthermore, the 4-domain model added clinically useful information. When frailty/vulnerability was decomposed, both medical burden and functional/cognitive impairment independently predicted lower LSM endorsement. In addition, replacing the social-support composite with its components did not reveal any “masked” or hidden social effect. All four social indicators remained uncertain, and all 95% HDIs crossed 1.00.

Sampling behavior for these subject-specific Bayesian models was excellent: 0 divergent transitions, 0 maximum tree-depth hits, BFMI approximately 0.70-0.80, maximum R-hat approximately 1.001, and minimum bulk ESS > 4,600. Response-level PSIS-LOO values were effectively indistinguishable across the sets (with elpd_loo values around -302 and SEs around 15).

The item ordering was also stable across all Rasch specifications: artificial nutrition was consistently the least accepted intervention, whereas ICU admission was consistently the most accepted.

A2.6 Bayesian marginal model with exchangeable within-person association

The Rasch latent-regression model is subject-specific: it estimates how predictors shift a person’s latent willingness to endorse LSM after accounting for person-level heterogeneity. As a complementary sensitivity analysis, we fit a Bayesian marginal model with exchangeable within-person association, which targeted a different estimand: the average endorsement probability in the sample (“population”). This model was built as a fully probabilistic joint Bayesian GEE-inspired sensitivity analysis (rather than a quasi-likelihood estimating equation). Its mean structure can be summarized as:

logit Pr(y_pi = 1) = alpha_i + X_p beta + u_pi,
with u_p· ~ MVN(0, sigma_u² R_exchangeable(rho))

Thus, the five responses from the same person are allowed to remain positively associated, but the model no longer assumes a single latent willingness parameter in the Rasch sense.

*Table A2.4*
*Bayesian marginal model with exchangeable within-person association across predictor specifications*

| **Specification** | **Predictor** | **OR** | **95% HDI** |
| --- | --- | --- | --- |
| **3-domain model** | Frailty/vulnerability | 0.43 | [0.26, 0.72] |
|  | Affective distress | 0.30 | [0.18, 0.53] |
|  | Social support | 1.12 | [0.70, 1.78] |
|  | Gender | 0.53 | [0.19, 1.44] |
|  | Self-responsibility | 0.45 | [0.15, 1.23] |
|  | Desired life expectancy | 1.48 | [0.92, 2.46] |
| **4-domain model (primary)** | Medical burden | 0.58 | [0.35, 0.93] |
|  | Functional/cognitive impairment | 0.57 | [0.34, 0.90] |
|  | Affective distress | 0.31 | [0.18, 0.53] |
|  | Social support | 1.12 | [0.71, 1.79] |
|  | Gender | 0.54 | [0.21, 1.48] |
|  | Self-responsibility | 0.46 | [0.16, 1.24] |
|  | Desired life expectancy | 1.46 | [0.90, 2.41] |
| **Social-as-single-indicators model** | Frailty/vulnerability | 0.44 | [0.27, 0.72] |
|  | Affective distress | 0.31 | [0.18, 0.53] |
|  | Gender | 0.53 | [0.19, 1.40] |
|  | Self-responsibility | 0.48 | [0.16, 1.27] |
|  | Desired life expectancy | 1.46 | [0.89, 2.38] |
|  | Social embeddedness | 1.31 | [0.82, 2.10] |
|  | Number of children | 1.19 | [0.75, 1.97] |
|  | Children living nearby | 0.86 | [0.53, 1.38] |
|  | Friendships | 0.93 | [0.59, 1.51] |

**Note.** Continuous predictors in the Bayesian models were entered on the 2-SD scale; ORs in this table have been back-transformed to represent a +1 SD increase in the original predictor: OR = exp(β/2). Binary predictors are reported as the 0-to-1 OR: OR = exp(β). Intervals are 95% HDIs.

The marginal models replicated the subject-specific Rasch models. Again, affective distress was the clearest negative predictor, and in the split-frailty specification both medical burden and functional/cognitive impairment remained independently negative. The social terms again remained uncertain.

These Bayesian marginal models were computationally heavier than the Rasch models, but still converged adequately for sensitivity purposes: 0 divergent transitions, 0 maximum tree-depth hits, BFMI approximately 0.73-0.81, maximum R-hat approximately 1.005, and minimum bulk ESS approximately 784. Response-level PSIS-LOO values again differed little across specifications, with elpd_loo values around -301 and SEs around 15, so they do not support a different model ranking.

A2.7 Indicator-level decomposition

Domain scores are useful because they facilitate (practical) interpretation and reduce the dimensionality of the predictor space. However, a legitimate concern is that aggregation might create an apparent effect that is not present at the raw-indicator level. We therefore also fit a fully disaggregated Bayesian subject-specific model in which the domain scores were replaced by the original indicators.

This decomposition analysis did not suggest that the domains manufactured the main result. Instead, the signal was already visible in the indicators themselves: the clearest negative contributions came from the Hamilton suicidality item and the Geriatric Depression Scale, whereas the social indicators remained weak and uncertain. Age and comorbidity remained negatively oriented, but with broader uncertainty once all original predictors competed simultaneously in the same model.

A2.8 Frequentist replications

The Bayesian models above answer the main scientific question, but it is still useful to ask whether the conclusions depend on Bayesian estimation. We therefore fit frequentist replications.

First, we fit a logistic generalized linear mixed model (GLMM) with item fixed effects and a patient random intercept. This is the maximum-likelihood counterpart of the subject-specific latent-response formulation. In plain language, it asks essentially the same conditional question as the Rasch model: which predictors shift each patient’s underlying tendency to endorse LSM, after accounting for stable between-person heterogeneity?

Second, we fit a logistic generalized estimating equation (GEE) with an exchangeable working correlation and robust sandwich standard errors. This is a population-averaged model: instead of focusing on a patient-specific latent tendency, it asks how predictors shift the mean endorsement probability across the sample.

*Table A2.5*
*Frequentist replication of the focal effects across predictor specifications*

| **Specification** | **Predictor** | **GLMM OR** | **GLMM 95% CI** | **GEE OR** | **GEE 95% CI** |
| --- | --- | --- | --- | --- | --- |
| **3-domain model** | Frailty/vulnerability | 0.33 | [0.19, 0.60] | 0.58 | [0.44, 0.77] |
|  | Affective distress | 0.22 | [0.12, 0.41] | 0.47 | [0.35, 0.63] |
|  | Social support | 1.09 | [0.64, 1.85] | 1.04 | [0.78, 1.38] |
| **4-domain model (primary)** | Medical burden | 0.49 | [0.28, 0.86] | 0.69 | [0.53, 0.92] |
|  | Functional/cognitive impairment | 0.49 | [0.28, 0.87] | 0.72 | [0.55, 0.94] |
|  | Affective distress | 0.22 | [0.12, 0.41] | 0.47 | [0.35, 0.63] |
|  | Social support | 1.09 | [0.64, 1.85] | 1.04 | [0.79, 1.38] |
| **Social-as-single-indicators model** | Frailty/vulnerability | 0.34 | [0.19, 0.61] | 0.58 | [0.44, 0.78] |
|  | Affective distress | 0.22 | [0.12, 0.40] | 0.45 | [0.33, 0.62] |
|  | Social embeddedness | 1.42 | [0.84, 2.42] | 1.21 | [0.93, 1.58] |
|  | Number of children | 1.21 | [0.68, 2.13] | 1.16 | [0.82, 1.64] |
|  | Children living nearby | 0.79 | [0.45, 1.38] | 0.86 | [0.64, 1.15] |
|  | Friendships | 0.87 | [0.51, 1.50] | 0.91 | [0.69, 1.19] |

**Note.** Continuous predictors in the frequentist replications were standardized conventionally, so ORs are directly interpretable as the change associated with a +1 SD increase. GLMM intervals are 95% Wald confidence intervals. GEE intervals are 95% robust Wald confidence intervals. Gender, self-responsibility, and desired life expectancy were included in all models but are omitted here for brevity because they remained imprecise throughout.

All focal conclusions replicated in the frequentist analyses. The GLMM aligned closely with the subject-specific Bayesian models, whereas the GEE showed the expected attenuation of ORs toward 1.00 because it targets the population-average rather than the patient-specific effect. Most importantly, however, the direction and pattern of effects remained the same: affective distress was consistently negative, the split-frailty model again showed independent negative contributions from medical burden and functional/cognitive impairment, and the social terms remained uncertain.

A2.9 Simplified continuous-score analysis

As a final low-assumption comparison, we converted the five LSM items into a standardized continuous factor score and regressed that score on the full set of original predictors using ordinary least squares with HC3 robust standard errors. This analysis does not model item-specific difficulty and the binary response structure and it asks whether the same broad directional pattern remains visible in an overly simplified representation of the outcome.

*Table A2.6*
*Simplified linear analysis of the standardized LSM factor score using the full indicator set*

| **Predictor** | **B** | **SE** | ***p*** |
| --- | --- | --- | --- |
| Charlson Comorbidity Index | -0.128 | 0.079 | .107 |
| Age | -0.135 | 0.076 | .077 |
| Sex (male = 1) | -0.237 | 0.167 | .158 |
| Self-responsibility in decision-making | -0.228 | 0.169 | .179 |
| Desired life expectancy | 0.020 | 0.074 | .792 |
| Hamilton suicidality item | -0.301 | 0.072 | < .001 |
| Social embeddedness | 0.088 | 0.074 | .237 |
| Number of children | 0.065 | 0.094 | .487 |
| Children living nearby | -0.045 | 0.080 | .576 |
| Friendships | -0.053 | 0.072 | .460 |
| Cognitive screening score | 0.142 | 0.088 | .108 |
| Geriatric Depression Scale | -0.181 | 0.071 | .012 |
| Barthel ADL | 0.013 | 0.081 | .872 |
| Instrumental ADL | 0.030 | 0.085 | .730 |

**Note.** Outcome = standardized one-factor LSM score (higher values indicate greater willingness to accept LSM). Coefficients are unstandardized regression coefficients on the standardized outcome. Standard errors are HC3 heteroscedasticity-robust. Model summary: R² = .344, adjusted R² = .278, F(14, 138) = 7.86, *p* < .001, condition number = 4.71.

The same affective pattern remained visible in this simplified analysis. The Hamilton suicidality item and the Geriatric Depression Scale were again the clearest negative predictors, whereas the social indicators remained unconvincing. Residuals showed modest deviation from normality, which is one reason the item-response models remain the inferential basis of the paper. Nonetheless, this simpler analysis points in the same substantive direction.

A2.10 Synthesis

Taken together, the robustness analyses support five conclusions.

1. The main result is not a prior artifact.
   Narrower shrinkage, wider shrinkage, heavier-tailed priors, and alternative scaling conventions all preserved the same substantive pattern.
2. Affective distress is the most robust predictor of lower LSM endorsement.
   This was shown in the Bayesian subject-specific models, the Bayesian marginal models, the frequentist GLMM, the frequentist GEE, and the simplified continuous-score analysis.
3. Frailty is more informative when decomposed.
   In the 4-domain specification, both medical burden and functional/cognitive impairment independently predicted lower willingness to accept LSM. The 3-domain frailty score captured the same general tendency, but the split model provides greater clinical information.
4. Social support does not show an independent association.
   This remained true whether social support was entered as a composite or as four separate indicators.
5. Changing the model family changes the estimand more than the conclusion.
   Subject-specific models yielded somewhat stronger associations than population-averaged models, but the direction and ranking of the effects remained the same.
